# Prevention of retinopathy of prematurity in preterm infants through changes in clinical practice and SpO_2_ technology

**DOI:** 10.1111/j.1651-2227.2010.02001.x

**Published:** 2011-02

**Authors:** Armando Castillo, Richard Deulofeut, Ann Critz, Augusto Sola

**Affiliations:** 1 Northeast Georgia Medical CenterGainesville, GA, USA; 2 Neonatal Service, Pediatrix Medical GroupDallas, TX, USA; 3 Division of Neonatal- Perinatal Medicine, Emory UniversityAtlanta, GA, USA; 4 Iberoamerican Society of NeonatologyMorristown, NJ, USA

**Keywords:** Hyperoxemia, Laser surgery, Newborns, Oxygen therapy, Prevention, Pulse oximetry saturation, Retinopathy of prematurity (ROP), Signal extraction technology (SET)

## Abstract

**Aim:**

To identify whether pulse oximetry technology is associated with decreased retinopathy of prematurity (ROP) and laser treatment.

**Methods:**

Inborn infants <1250 g who had eye exams were compared at two centres in three periods. In Period 1, SpO_2_ target was ≥93% and pulse oximetry technology was the same in both Centres. In Period 2, guidelines for SpO_2_ 88–93% were implemented at both centres and Centre B changed to oximeters with signal extraction technology (SET®) while Centre A did not, but did so in Period 3. One ophthalmology department performed eye exams using international criteria.

**Results:**

In 571 newborns <1250 g, birth weight and gestational age were similar in the different periods and centres. At Centre A, severe ROP and need for laser remained the same in Periods 1 and 2, decreasing in Period 3–6% and 3%, respectively. At Centre B, severe ROP decreased from 12% (Period 1) to 5% (Period 2) and need for laser decreased from 5% to 3%, remaining low in Period 3.

**Conclusion:**

In a large group of inborn infants <1250 g, a change in clinical practice in combination with pulse oximetry with Masimo SET, but not without it, led to significant reduction in severe ROP and need for laser therapy. Pulse oximetry selection is important in managing critically ill infants.

## Introduction

Retinopathy of prematurity (ROP) is a devastating and common blinding disease in developed and developing countries (1,2). However, most cases of ROP are mild and regress spontaneously. The incidence of this disease has been reported to be as high as 29% in very low birth weight infants (VLBW) and is strongly associated with smaller and sicker infants (3–5). There are many risk factors involved in the pathogenesis of ROP (5–8), and two phases of abnormal vessel development have been described in ROP. Phase I is characterized by delayed retinal vascular growth and partial regression of existing vessels. Phase II is characterized by pathological vessel growth associated with hypoxia (2). The use of oxygen and fluctuations of arterial oxygen are considered major risk factors for the development severe ROP in preterm infants. (2,6,8). We and others have shown that lower saturation targets are associated with improved rates of ROP (9–14) and that there is a treatment-by-gender effect when aiming to avoid hyperoxia in preterm infants in the Neonatal Intensive Care Unit (NICU) (15). Strategies to lower the incidence of ROP include the following: education and commitment of bedside care providers, guidelines to decrease hyperoxemic periods and wide changes in oxygenation and the advances in saturation monitoring technology. The impact of each of these interventions on the incidence of ROP is not easy to discriminate. In the last decade, it has become clear that performance of pulse oximeters differ by brand, with some brands having a much higher incidence of false alarms and holding periods and reduced reliability (16–21). Of the next generation pulse oximetry technologies, only one has signal extraction technology (SET), which has been shown to have fewer false alarms and missed events in challenging clinical situations such as during motion and low perfusion. (22–26). However, no studies have explored the role that SpO_2_ technology plays in the prevention or ROP. We designed this study to determine whether the incidence of severe ROP is associated with the type pulse oximetry technology utilized in the NICU, when oxygen saturation limits are aimed to be maintained between 88 and 93% after education and guidelines are implemented. Our hypothesis was that the relative risk reduction in severe retinopathy of prematurity is associated with the saturation technology utilized in neonatal intensive care units.

## Patients and Methods

After Internal Review Board approval, this observational descriptive study was conducted in two centres at Emory University during two consecutive 3 -year periods (Periods 1 and 2) and an 18- month follow-up period (Period 3). Centre A was Grady Memorial Hospital, and Centre B was Emory Crawford Long Hospital. Table 1 shows a schematic representation of the study design. Inborn infants, with birth weight <1250 g that had detailed ROP examinations by an ophthalmologist from Emory University following AAP and AAO guidelines (27), were included in this study. The exclusion criteria were as follows: newborns with major congenital anomalies, outborn infants, infants with birth weight ≥1250 g and those who had no ophthalmologic examination.

**Table 1:** Schematic description of the study design

	Period I3 years2001–2003	Period II3 years2004–2006	Period III18 months2007–2008
SpO_2_ Range	SpO_2_ ≥92%	SpO_2_ 88–93%	SpO_2_ 88–93%
Pulse Oximetry Type	Centres A and B: Nellcor®	Centre A: Nellcor®Centre B: Masimo SET	Centres A and B: Masimo SET®
Practice Intervention	None	Educational process, guidelines development and implementation	
Practitioners and data	Leadership and health care team members: same (but for RN's and RRT's; see text). Data from the two centres collected prospectively into same data base		

During the first period, oxygen saturation targets were between 92% and 100%, and the pulse oximeters used at both centres were Nellcor N-300 and Nellcor N-395, (Nellcor®, Covidien, Boulder, CO, USA). As part of an intense quality improvement effort, an educational process was initiated for oxygen utilization and monitoring in the two centres. This led to guideline development and implementation, as described previously (9,10). In Period 2, the new practice guidelines for lower oxygen saturation targets of 88–93% for premature infants receiving supplemental oxygen were implemented at both centres (Table 1). During Period 2, the pulse oximeters were changed to Masimo SET® (Masimo, Irvine, CA, USA) in Centre B while Centre A continued to use the Nellcor® technology. During Period 3, both centres continued to practise the guidelines for oxygen saturation targets and Centre A changed to pulse oximetry with SET so that both centres were using SET technology. During all three periods, the medical leadership, attending neonatologists, neonatal fellows and neonatal nurse practitioners were the same in both centres and followed the same guidelines for the care of neonates (Table 1). Of course, other improvements other than oxygen management did occur over the study period, but they affected at all infants regardless of the type of monitoring used.

### Data analyses and statistics

In both centres, the data were collected prospectively and entered into a single, combined database by the same personnel for subsequent analysis. The data for all infants in both centres were also reported to Vermont Oxford Network (VON). Statistics included chi square, Student’s *t*-test and ANOVA when appropriate. Only infants with detailed ROP examinations were used in the denominator to calculate the incidence of severe ROP and need for laser therapy in all periods and centres. All the statistical analyses were carried out using the same software (SPSS version 15.0 for Windows; SPSS, Chicago, IL, USA).

## Results

During the three study periods, there were 774 newborns with BW <1250 g admitted to the study centres. Of them, 571 had eye exams and met all the inclusion criteria. From Centre A, there were 138 infants included during Period 1, 113 during Period 2 and 65 during Period 3. From Centre B, there were 83 infants included in Period 3, 115 infants in Period 2 and 57 in Period 3. There were no significant differences in patient characteristics between the two centres. Table 2 summarizes some of the characteristics of the patient population. During the three study periods, we did not identify any child who suffered from stage V ROP in either of the centres.

**Table 2:** Demographics of the studied population

	A			B		
		
Centres Period (n)	Period 1 (138)	Period 2 (113)	Period 3 (65)	Period 1 (83)	Period 2 (115)	Period 3 (57)
Gestational weeks	27 ± 2	27 ± 2.3	27 ± 1.6	26.8 ± 2.1	26.8 ± 2.2	27 ± 2.4
Birth Weight (g)	922.8 ± 190	914 ± 208	907 ± 183	889.6 ± 203	866 ± 198	897 ± 206
Antenatal Steroids	78%	79%	80%	64%	77%	81%
Female	56%	51%	53%	55%	53%	52%
Mechanical Ventilation	92%	83%	86%	85%	81%	83%
Small for gestation	21%	21%	20%	21%	23%	21%

No significant statistical significance was identified for any variable.

The rate of ROP III and IV and laser treatment in examined infants with birth weight <1250 g in Centre B were 12% and 5% in Period 1 and significantly decreased to 5% and 3% in Period 2 following the change to SET technology (p < 0.05) (Table 3). Based on this decrease in the incidence of severe ROP in Centre B, the relative risk reduction can be calculated at 58%, with a number needed to treat of 14. In Centre B, the incidence remained low in Period 3, 4% and 2%, respectively. In Centre A in Period 1, ROP III–IV and laser rates were 13% and 4.5%, respectively, and did not decrease in Period 2. In Period 3, following the change to SET technology, ROP III–IV decreased to 6% with a relative risk reduction of 54% (p < 0.05), and laser rates decreased to 3%.

**Table 3:** Rates of retinopathy of prematurity (ROP) and laser surgery for each centre in each period in examined infants <1250 g

	Centre A			Centre B		
		
	Period 1	Period 2	Period 3	Period 1	Period 2	Period 3
ROP III–IV	13%	13%**,†	6%†	12%¶	5%**,¶	4%
LASER	4.5%	5%	3%	5%*	3%*	2%

All comparisons (†;**; ¶; *): p < 0.05.

We also examined the individual incidence of severe ROP for each of the years of Periods 1 and 2 at each centre. As expected, there was variability between each year. At Centre A, the incidence of ROP III–IV varied between 15% and 10% during the years of Period 1 and was relatively stable with an average of 13% per year during Period 2 while the technology was not changed. Similarly, at Centre B, the incidence of severe ROP varied during the years of Period 1 (10–13%). Unlike Centre A, severe ROP decreased each year during Period 2, starting at 10% in the first year and decreasing to 3% in the last year of the period, The rates of laser surgery treatment for severe ROP in Centre A decreased during Period 3 while at Centre B the rates decreased during Period 2 and remained as low during Period 3. With ‘lower’ oxygen saturation targets of 88–93%, the mortality was similar and there was no increase in periventricular leucomalacia; the rate of bronchopulmonary displasia was improved, and the incidence of necrotizing enterocolitis and patent ductus arteriosus did not increase. Additionally, the longer term outcome was better in the period with lower targets in the infants available for follow up (10).

## Discussion

This study confirms that while ROP is a potentially serious condition, it can be reduced in at risk newborns. The findings of this study show a significant decrease in the incidence of severe ROP and need for laser surgery during the period after education and practice changes when SET pulse oximetry technology was used. Furthermore, the lower incidence of ROP following the practice changes and change in pulse oximetry was progressively more significant year after year. In this study of 571 infants, none was blinded by stage V ROP and only about ≤5% needed treatment to prevent blindness.

Among the most important ROP, prevention efforts are to decrease wide changes in oxygenation and hyperoxemic periods (9–16,28). At the two centres studied, the same intense educational process and clinical guidelines aimed to decrease hyperoxemia, and wide changes in oxygenation were implemented. In 1992, Flynn et al. were the first to describe an association between the incidence and severity of retinopathy of prematurity and the duration of exposure to arterial oxygen levels of 80 mmHg or higher, measured transcutaneously (29). As we have shown that saturations >94% are likely to be associated with PaO_2_ > 80 mmHg in infants breathing supplemental oxygen (30), the target values were to avoid those levels of saturation. The leadership, physicians and nurse practitioners were the same at both centres during the three study periods. The pulse oximetry technology changed to SET technology at one centre after Period 1 and stayed the same at the other centre but then was changed at the second centre during Period 3. The findings of this study show a strong positive association between the use of pulse oximetry with SET and a reduction in the incidence of ROP.

Many factors contribute to good medical care and outcome improvement. Among them are education, training, commitment, team work, eradicating bad practices, and the correct use of the best available technology. In general, it is well accepted in most areas of medical care that technology by itself, without education and appropriate use, serves very little value for care improvement. On the other hand, well-trained committed clinicians obtain worse outcomes when using technology that is obsolete, outdated or of lesser quality. The findings clearly suggests that, when all other interventions are the same, an educational process and improved care guidelines along with a change to improved pulse oximetry technology are useful for reducing the incidence of severe neonatal ROP and need for laser surgery.

In contrast to the interventional devices and therapies, there is a tendency by some clinicians to believe that non-therapeutic monitoring devices with similar functionality deliver similar performance. However, as clinicians, we all understand that the decisions we make, right or wrong, are often based on the numbers shown on patient monitors. Therefore, the accuracy and reliability of monitored physiologic information can be just as important, if not more important, than the therapeutic interventions that follow. In this study, we have shown that use of one type of pulse oximetry technology appears to strongly affect the incidence of ROP.

We acknowledge that there are some limitations in this study. This was not a randomized controlled trial, so the results cannot definitively isolate the pulse oximetry technology as the major only factor contributing to decreasing incidence of ROP. However, it is unlikely that such a study could ever be performed in a randomized way. In addition, while the physicians and nurse practitioners were the same at both centres, the bedside nurses and respiratory therapists were different. As the rates of severe ROP were significantly lower at Centre B compared to Centre A in period 2 (Table 3) in association with implementation of SET technology and a reduction in severe ROP and need for laser surgery occurred in Centre A in Period 3, when change of technology occurred, it is unlikely that bedside nurses and respiratory therapists played a major role in the different outcomes during the different periods. Nonetheless, the significance or ‘weight’ of different bedside health care providers cannot be separately discriminated in this study.

Finally, a very recent article reports markedly decrease ROP but worse mortality when saturation targets are 85–89% in very tiny infants. In the current and in all other studies published to date, no one has aimed for a target of 85–89%. Therefore, we cannot compare or comment on those findings. Further data are waited from other large ongoing randomized clinical trials.

## Conclusions

In this study of inborn infants with birth weight <1250 g treated by the same physicians and nurse practitioners using the same clinical guidelines aimed at decreasing hyperoxemia and wide changes in oxygenation, a reduction in the incidence of severe ROP and need for laser therapy were associated with the use of signal extraction pulse oximetry. The findings lend further support to the significance of using improved saturation monitors in managing critically ill infants. Education, guidelines and practice changes for reducing ROP may be more effective when accurate and reliable pulse oximetry technology is used.

## References

[1] Tasman W, Patz A, McNamara JA, Kaiser RS, Trese MT, Smith BT (2006). Retinopathy of prematurity: the life of a lifetime disease. Am J Ophthalmol.

[2] Chen J, Smith LE (2007). Retinopathy of prematurity. Angiogenesis.

[3] Shah VA, Yeo CL, Ling YL, Ho LY (2005). Incidence, risk factors of retinopathy of prematurity among very low birth weight infants in Singapore. Ann Acad Med Singapore.

[4] Delport SD, Swanepoel JC, Odendaal PJ, Roux P (2002). Incidence of retinopathy of prematurity in very-low-birth-weight infants born at Kalafong Hospital, Pretoria. S Afr Med J.

[5] Saunders RA, Donahue ML, Christmann LM, Pakalnis AV, Tung B, Hardy RJ (1997). Racial variation in retinopathy of prematurity. The Cryotherapy for Retinopathy of Prematurity Cooperative Group. Arch Ophthalmol.

[6] Smith LE (2003). Pathogenesis of retinopathy of prematurity. Semin Neonatol.

[7] Karna P, Muttineni J, Angell L, Karmaus W (2005). Retinopathy of prematurity and risk factors: a prospective cohort study. BMC Pediatr.

[8] York JR, Landers S, Kirby RS, Arbogast PG, Penn JS (2004). Arterial oxygen fluctuation and retinopathy of prematurity in very-low-birth-weight infants. J Perinatol.

[9] Chow LC, Wright KW, Sola A, for the CSMC Oxygen Administration Study Group (2003). Can changes in clinical practice decrease the incidence of severe retinopathy of prematurity in very low birth weight infants?. Pediatrics.

[10] Deulofeut R, Critz A, Adams-Chapman I, Sola A (2006). Avoiding hyperoxia in infants ≤ 1,250 g is associated with improved short- and long-term outcomes. J Perinatol.

[11] Wright KW, Sami D, Thompson L, Ramanathan R, Joseph R, Farzavandi S (2006). A physiologic reduced oxygen protocol decreases the incidence of threshold retinopathy of prematurity. Trans Am Ophthalmol Soc.

[12] Sears JE, Pietz J, Sonnie C, Dolcini D, Hoppe G (2009). A change in oxygen supplementation can decrease the incidence of retinopathy of prematurity. Ophthalmol.

[13] Chen ML, Guo L, Smith LE, Dammann CE, Dammann O (2010). High or low oxygen saturation and severe retinopathy of prematurity: a meta-analysis. Pediatrics.

[14] SUPPORT Study Group (2010). Target ranges of oxygen saturation in extremely preterm infants. NEJM.

[15] Deulofeut R, Golde D, Augusto S (2007). Treatment-by-gender effect when aiming to avoid hyperoxia in preterm infants in the NICU. Acta Paediatr.

[16] Ahlborn V, Bohnhorst B (2000). False alarms in very low birthweight infants: comparison between three intensive care monitoring systems. Acta Paediatr.

[17] Bohnhorst B, Peter CS, Owitz CF (2000). Pulse oximeters’ reliability in detecting hypoxemia and bradycardia: comparison between a conventional and two new generation oximeters. Crit Care Med.

[18] Malviya S, Reynolds PI, Voepel-Lewis T, Siewert M, Watson D, Tait AR (2000). False alarms and sensitivity of conventional pulse oximetry versus the Masimo SET technology in the pediatric postanesthesia care unit. Anesth Analg.

[19] Poets CF, Urschitz MS, Bohnhorst B (2002). Pulse oximetry in the neonatal care unit. Detection of hyperoxemia and false alarm rates. Anesth Analg.

[20] Hay WW, Rodden DJ (2002). Reliability of conventional and new oximetry in neonatal patients. J Perinatol.

[21] Durbin CG, Rostow SK (2002). Advantages of new technology pulse oximetry with adults in extremis. Anesth Analg.

[22] Durbin CG, Rostow SK (2002). More reliable oximetry reduces the frequency of arterial blood gas analysis and hastens oxygen weaning after cardiac surgery: a prospective, randomized trial of the clinical impact of the new technology. Crit Care Med.

[23] Murthy LCT, Goyal RM (2005). Masimo – a new reliable noninvasive method of detecting oxygen saturation in critically ill. Indian J Anaesth.

[24] Torres A, Skender KM (2004). Pulse oximetry in children with congenital heart disease: effects of cardiopulmonary bypass and cyanosis. J Intensive Care Med.

[25] Sahni R, Gupta A, Ohira-Kist K, Rosen TS (2003). Motion resistant pulse oximetry in neonates. Arch Dis Child Fetal Neonatal Ed.

[26] Kawagishi T, Kanaya N, Nakawama M, Kurosawa S, Namiki A (2004). A comparison of the failure times of pulse oximeters during blood pressure cuff-induced hypo perfusion in volunteers. Anesth Analg.

[27] American Academy of Pediatrics (2001). Section on Ophthalmology, American Academy of Ophthalmology, and American Association for Pediatric Ophthalmology and Strabismus. Screening examination of premature infants for retinopathy of prematurity. Pediatrics.

[28] Sola A, Saldeño YP, Favareto V (2008). Clinical practices in neonatal oxygenation: where have we failed? What can we do?. J Perinatol.

[29] Flynn JT, Bancalari E, Snyder ES (1992). A cohort study of transcutaneous oxygen tension and the incidence and severity of retinopathy of prematurity. NEJM.

[30] Castillo A, Sola A, Baquero H, Neira F, Alvis R, Deulofeut R (2008). Pulse oxygen saturation levels and arterial oxygen tension values in newborns receiving oxygen therapy in the neonatal intensive care unit: is 85% to 93% an acceptable range?. Pediatrics.

